# Comparing Sexual Function and Quality of Life in Polycystic Ovary Syndrome and Healthy Women 

**Published:** 2016-06

**Authors:** Vida Shafti, Sara Shahbazi

**Affiliations:** 1 School of Medicine, Tonekabon Islamic Azad University, Tonekabon, Iran; 2 Department of Literature and Humanities, School of Psychology, Guilan University, Rasht, Iran

**Keywords:** Sexual Function, Quality of Life, Polycystic Ovary Syndrome (PCOS)

## Abstract

**Objective:** Polycystic ovary syndrome (PCOS) is one of the most common endocrine disorders that is associated with different metabolic, reproductive and psychological consequences. The main aim of this study was to compare the sexual function and quality of life in women with polycystic ovary syndrome and healthy women.

**Materials and methods:** This is a causal-comparative study in which 129 women with polycystic ovary syndrome were qualified as the research group. The control group consisted of 125 healthy women. The sampling method was convenient and was done using Rotterdam criteria. Women of both research and control groups responded to the FSFI and WHOQOL-BREF questionnaires. Data were analyzed with SPSS software using MANOVA.

**Results:** According to findings, all of quality of life subscales except environment domain were significantly lower in research group than healthy group (p < 0.01), but none of sexual function subscales were significantly different between two groups (p > 0.05).

**Conclusion:** Women with PCOS in term of some quality of life parameters have lower performance than healthy women. Therefore, it seems to be essential to increase awareness about symptoms and psychological consequences and referring process in order to take advantage of the advisory services.

## Introduction

Polycystic ovary syndrome (PCOS) is one of the most prevalent endocrine disorders resulting numerous impacts on physical and psychological aspects of an individual including hirsutism, Cystic acne, obesity, hair loss, Weight gain (1, 2), higher chance of certain diseases such as type II diabetes, endometrial cancer, High cholesterol, cardiovascular problems (3-5) and also anxiety and emotional stress (6) that can be the result of changes of appearance, menstrual irregularities, and possible disturbances in the sexual attitude and behavior which may end in an ambiguous gender identity (7). This syndrome is widely prevalent in a way that it can be said one out of every 15 women around the world is dealing with PCOS (8).

Sometimes this syndrome causes high levels of anxiety and tension that lead to depression, eating disorders, sexual dysfunction and so forth (9). Decreased feminity, negative self-image, depression and increased male hormones are observed in these women and may influence their sexual function.

Sexual function is a multi-dimensional issue which is under influence of psychological, biological and interpersonal factors (10) and it means how a person accomplishes a sexual respond cycle. This cycle is a four-phase physiologic process including excitement (arousal), plateau, orgasm and resolution and is done by complicated interaction between psychological, social, environmental and biologic (hormonic, vascular, muscular and neurotic) factors (11). Some studies have shown that this syndrome impairs women's sexual function (12) but it should be considered that in sexual functioning cultural, religious, racial and ethnical factors are also included (13, 14). Most of the studies have reported sexual malfunction of women with PCOS in vaginal lubrication and excitation (15). Sometimes these women's sexual function has been reported lower than normal women in domain of excitation, eroticism and orgasm (16). Mood disorders and side effects of some medications make these women vulnerable sexual function wise (17, 18). But there are some studies that have not confirmed the sexual function impairment in these women. The results are paradoxical as several studies have reported that women with PCOS have gained similar score as normal women in sexual function tests (19, 20). Månsson et al. (21) have studied the impact of this syndrome on women's sexual function and they have found that despite lower life satisfaction among them, but weight gaining as one symptom of this syndrome does not have a considerable influence on their sexual function and even increased testosterone has a positive correlation with their sexual function. Anyway, all physical, psychological and social problems which these women are dealing with can reduce their quality of life just like what different studies have confirmed (22-24).

Although quality of life points out the rate of how people feel about their physical, emotional and social abilities and it is an individual concept (25), but based on the definition provided by The World Health Organization (26), quality of life can be defined as a state of complete physical, mental, and social wellbeing and four dimensions are considered for it including: Physical health (pain and feeling discomfort, sleep and resting and ability to take care of everyday functions); psychological aspect (appearance, positive and negative emotions, memory, concentration and self-confidence); Social contacts (personal contacts, social support and sexual activities) and social environment (financial security, home environment, information access, participation in social activities and transportation facilities). Studies have confirmed that quality of life is dramatically under influence of health status especially as chronic diseases have undesirable impacts on individual's social, psychological and physical status (27). In some researches, quality of life of women with PCOS has been reported lower than of normal women (28, 29) and even lower than of people with some diseases such as diabetes, asthma and epilepsy (30).

Polycystic ovary syndrome is prevalent worldwide and has numerous negative consequences especially as it is closely associated with reproduction reduction and infertility (31) which concerns women with PCOS and their relatives. Therefore there are a lot of studies performed in this domain. As much as these negative consequences be elucidated, it will help to direct treatment to the correct path and improve these women's quality of life. So the aim of this study is to compare sexual function and quality of life of women with PCOS with normal women and it is hypothesized that the sexual function and quality of life of women with PCOS is lower in comparison with normal women.

## Materials and methods

This causal-comparative study was performed with participation of 129 married women with polycystic ovary syndrome and 125 healthy married women within the age range of 18-45 who visited Shahid Rajaee Hospital and selected women infertility clinics in Tonekabon between November 2013 to June 2014. The sample cases were selected by Convenience sampling method. Women who their diagnosis of PCOS had been medically verified, and were intended to participate were included in the study. Control group were also consistent of 125 healthy married women within the age range of 18-45 who had no chronic disease, had regular menstrual cycle and they were selected from clinic's employees and patients' companions by Convenience sampling method. At last, after excluding the not-completely-filled questionnaires, 129 women with PCOS and 125 healthy women were included in the study. 

All participants of experimental group (women with PCOS) and control group (Healthy women) had chosen to enter the study well-informedly. They were assured about confidentiality of collected data and their verbal acceptance about study conditions were collected before entering the study. Exclusion criteria (experimental and control group) were including: Diagnosed with psychological disorders or use of psychiatric medications or history of hospitalization in Neurology and psychiatry ward, Pregnancy or lactation, any chronic disease (Diabetes, endocrine disorders, receiving treatment for PCOS in the previous two months)*.* Intrusion and exclusion of participants of experimental group were decided by specialist's diagnosis, medical records and individual's self-report and for control group it was done based on individual's self-report. Later, selected participants have filled sexual function and WHO quality of life (26-question brief form) questionnaires. Data analysis was performed by SPSS ver. 20 and Multivariate analysis of variance (MANOVA). 

Female sexual function index (FSFI): It is a six-dimensional 19-item questionnaire designed by Rosen et al (32) to assess women's sexual function in desire, arousal, lubrication, orgasm, satisfaction and pain. 1 to 5 scores are assigned for every question in desire dimension, and for other dimensions 0 to 5 scores are determined. Higher total score indicates better sexual function. Retest reliability coefficient of this questionnaire has been obtained .79 to .86 and Cronbach's alpha has been estimated .82 or above (32). In present study, the inner consistency was measured .95 by Cronbach's Alpha method.

Brief form of The World Health Organization quality of life questionnaire (WHOQOL-BREF): This instrument has been designed and translated in 11 countries simultaneously and assesses four aspects of physical health, mental health, social relationships, and environment by 26 items (33). Skevington's (34) findings have confirmed high inner consistency of this questionnaire which has been calculated .92 by Cronbach's Alpha. The Cronbach's Alpha of this scale in present study has been measured 0.83.

## Results

Totally 125 participants (49.2%) of sample group were healthy women with mean of age of 32.79 years old and 129 participants (50.8%) were patients with PCOS with mean of age of 30.10. The total participants' mean of age was 31.42 years old. There were 52 persons (20.5%) under graduated, 93 persons (36.6%) graduated from high school, 17 persons (6.7%) with Associate’s degree, 58 persons (22.8%) with Bachelor’s degree and 12 persons (4.7%) with Master's degree.

Findings of Levene’s test (Table 2) have shown that any of variables of sexual function and quality of life are not significant (p > 0.05), so the hypothesis of variance homogeneity is observed. Kolmogorov–Smirnov test findings have also indicated that all sexual function and quality of life subscales are complying with assumption of normality (p > 0.05). However, assessment of data characteristics had shown that the assumption of consistent variance-covariance matrix (p < 0.001, Box's M = 50.41) is not confirmed, so the Pillai's trace had been used to assess the significance of multivariate impact.

Pillai's trace had shown that the group effect on linear combination of dependent variables is not significant (F = 0.93, p > 0.05 and Partial n^2^ = 0.02). In other words, there is not a significant difference between healthy group and patients group in any of sexual function factors. Findings (Table 3) have indicated that mean of scores gained by healthy women and the ones with PCOS haven't reached significance level in any of sexual function factors (p > 0.05).

The analysis of characteristics of data had also indicated that the statistic assumption of consistent variance-covariance matrix is not confirmed for quality of life factors (M = Box's 70.08, p < 0.001), so the Pillai's trace had been used to assess the significance of multivariate impact. 

**Table 1:** Mean and Standard deviation of sexual function and quality of life in two groups

**Variable**	**Healthy**		**PCOD**	
	
	**Mean**	**SD**	**Mean**	**SD**
Desire	6.55	1.68	6.18	1.56
Arousal	13.38	4.73	12.74	3.92
Lubrication	13.92	4.64	13.72	4.13
Orgasm	11.53	4.12	10.81	3.55
Satisfaction	12.19	4.18	11.40	3.75
Pain	10.80	4.16	10.57	3.82
Physical Health	26.98	3.74	25.37	3.94
Mental Health	22.79	2.88	21.59	3.41
Social Contacts	12.05	1.82	11.24	2.19
Social Environment	28.77	3.90	27.61	5.37

**Table 2:** Mean and Standard deviation of sexual function and quality of life in two groups

	**F**	**df1**	**df2**	**p value**
Desire	1.04	1.68	6.18	1.56
Arousal	1.89	4.73	12.74	3.92
Lubrication	0.58	4.64	13.72	4.13
Orgasm	1.92	4.12	10.81	3.55
Satisfaction	0.06	4.18	11.40	3.75
Pain	0.22	4.16	10.57	3.82
Physical Health	0.24	3.74	25.37	3.94
Mental Health	2.81	2.88	21.59	3.41
Social Contacts	1.80	1.82	11.24	2.19
Social Environment	0.24	3.90	27.61	5.37

Pillai's had shown that the group effect on linear combination of dependent variables is significant (F = 3.40, p < 0.05 and Partial n^2^ = 0.05). In other words, there is a significant difference between healthy group and patients group in at least one of the quality of life factors.

Statistic of One-way ANOVA had been performed for every dependent variable separately to determine the source of significance of multivariate effect. Table 4 shows that group significantly effects physical health (F (1, 244) = 10.86, p < 0.01 and Partial n^2 ^= 0.04), Mental health (F (1, 244) = 8.70, p < 0.01 and Partial n^2 ^= 0.03) and Social contacts (F (1, 244) = 10.00, p < 0.01 and Partial n^2^ = 0.03). The group effect on social environment is not statistically significant (F (1, 244) = 3.76, p > 0.05 and Partial n^2 ^= 0.01).

## Discussion

Polycystic Ovary syndrome is known as a prevalent disorder among women that leads to serious negative impacts on their different aspects of life (1, 2). This study has shown that although the quality of life of women with PCOS is significantly poorer in comparison with normal women, but there is no significant difference in their sexual function which the reason has to be discussed. All dimensions of these women's life including physical health, social contacts and mental health are significantly different from normal people except in social environment in which no significant difference has been observed. Several studies have confirmed the correlation between quality of life and polycystic Ovary syndrome (24, 28, 29). Of all these physical problems, Weight gain is known as one of the most serious symptom of this syndrome which may increase the risk of other diseases such as diabetes and cardiovascular diseases (35). Furthermore, as these women deals with numerous problems including infertility, they may also experience high level of stress and anxiety (31) which can threaten their physical health as well. On the other hand, several studies have determined that there is a significant positive correlation between stress and chronic physical diseases (36, 37) which this issue can also weaken these women's physical health.

**Table 3:** Results of variance analysis test of sexual function

	**SS**	**df**	**MS**	**F**	**p**
Desire	8.47	1	8.47	3.19	0.07
Arousal	25.08	1	25.08	1.32	0.25
Lubrication	2.33	1	2.33	0.12	0.72
Orgasm	31.99	1	31.99	2.15	0.14
Satisfaction	36.98	1	36.98	2.34	0.12
Pain	3.38	1	3.38	0.21	0.64

**Table 4:** Results of variance analysis test of quality of life

	**SS**	**df**	**MS**	**F**	**p**
Physical Health	160.98	1	160.98	10.86	0.00
Mental Health	87.84	1	87.84	8.70	0.00
Social Contacts	40.65	1	40.65	10.00	0.00
Social Environment	83.12	1	83.12	3.76	0.05

Other problems that women with this syndrome are dealing with such as Acne, hirsutism, hair loss, and Weight gain can also damage their self-esteem about their appearance and regarding the importance role of appearance in women's self-confidence, it can limit their social activities (38). Self-esteem is necessary for successful social relations and studies have also confirmed that people with higher self-esteem are the ones who have healthier social contacts (39). Considering high risk of possible infertility in these women, they also face family problems and losing their friends' support.

The present study confirms that mental health level is also lower in women with polycystic ovary syndrome in comparison with normal women. Several studies have reported that these women's mental health is at risk and there are depression, anxiety and other psychological problem seen among them (9). In fact, like other chronic diseases, this syndrome involves patients in complicated treatment process as well and leads them to burnout and disappointment. However, this study has not found any significant difference between social environment of women with and without PCOS, but it cannot be said for sure and the quality of social environment of each patient has to be considered. Social environment includes financial issues, transportation and home environment. It has to be considered that besides the burden of high expenses of treatment on families, patients has to spend a lot of time on clinical visits and it may influence their duties and profession and challenge their social environment. These impacts on their life took time to be seen, so they should not be ignored. Certain studies emphasize on these women's quality of life and consider its changes in treatment process (36).

As it was said before, in this study, no difference has been measured between any dimensions of sexual function (desire, arousal, lubrication, orgasm, satisfaction and pain) of women with PCOS and healthy ones. This study is in accordance with Ferraresi et al. (19) and Stovall et al. (20) while Hahn et al. (12), Esposito et al. (17) and Trent et al. (18) have not confirmed findings of this study. In fact, there are a lot of paradoxes in available studies. The reason of these paradoxical findings can be cultural, religious, age differences and also different duration of disease and this issue have to be studied more specifically. Anyway it has to be mentioned that despite all problems in appearance and weight gain that these women are facing, but the increased testosterone level can help them to have a good sexual function as Manson et al. (21) have found that higher level of testosterone in these women has positive correlation with their sexual function. However this finding also emphasize that although there are negative impacts of this syndrome on self-confidence and increased tension and anxiety, but it has to be mentioned that sexual function is more physiologic than psychological and in fact it means how a person accomplish physiologic cycle of a sexual response (excitement (arousal), plateau, orgasm and resolution) (11). However the role of mental status cannot be ignored but it seems that it is not that much important. Even though, if based on those studies which have reported poorer sexual function in women with PCOS rather that normal women, we assume that mood problems, depression, anxiety and lack of self-confidence impair these women's sexual function, but still their mental health have to be analyzed more specifically and it has to be considered that sexual function impairment can be intensified as disease progresses or because of side effects of used medications.

There were some boundaries in the way of this studies including: lack of specialized centers specially infertility clinics specifically for polycystic ovary syndrome in Tonekabon which had limited the access to women with this syndrome, lack of information about the necessity of performing such studies on certain disorders such as PCOS which is not considered as a disease by lot of people and treat it as a private and personal matter , denying certain physical and psychological symptom of this syndrome especially in appearance and beauty domain. This study suggests that it seems mandatory to refer patients with PCOS to psychologists and consultants to improve their psychological indexes such as quality of life and to held workshops and provide training packages by health system and specialists in this domain to increase public knowledge on this syndrome and enhance their quality of life.

## Conclusion

Women with PCOS in term of some quality of life parameters have lower performance than healthy women. Therefore, it seems to be essential to increase awareness about symptoms and psychological consequences and referring process in order to take advantage of the advisory services.
